# Supplementary material for: Effect of Pressure Conditions in Uterine Decellularization Using Hydrostatic Pressure on Structural Protein Preservation
Source: Bioengineering (Basel). 2023 Jul 7;10(7):814. doi: 10.3390/bioengineering10070814 (PMC10376797; doi:10.3390/bioengineering10070814)
Supplement: Supplementary file 1 [file bioengineering-10-00814-s001.zip › bioengineering-2440627-SI.pdf]

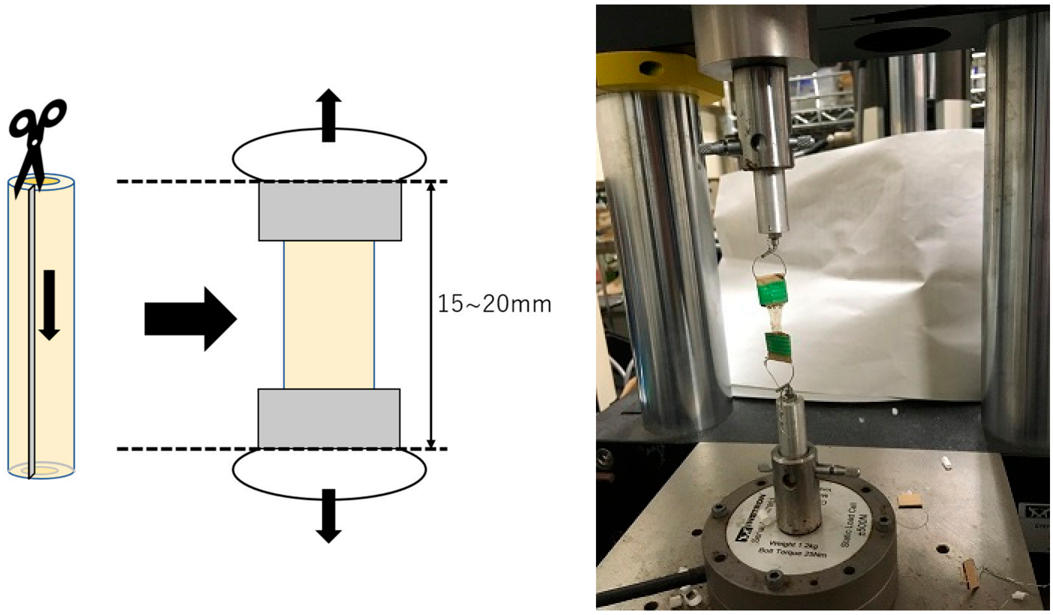

Figure S1. Tensile test. Schematic of sample preparation for uniaxial tensile tests (left) and the fixation to the probe of the testing machine (right)

Young's modulus was calculated based on the Voigt viscoelastic model:

$$\sigma = E\varepsilon + \eta C_1$$

E: Young's modulus; η: viscosity; σ: stress; ε: strain; C_1: strain speed

Equation S1. Calculation of Young's modulus

Stress-strain curves are recorded automatically by the testing machine. Strain speed is calculated from the constant displacement speed. Young's modulus is obtained via curve fitting of the stress-strain curves.
